# The burden of out of pocket costs and medical debt faced by households with chronic health conditions in the United States

**DOI:** 10.1371/journal.pone.0199598

**Published:** 2018-06-25

**Authors:** Patrick Richard, Regine Walker, Pierre Alexandre

**Affiliations:** 1 Department of Preventive Medicine and Biostatistics, Uniformed Services University of the Health Sciences (USU), Bethesda, Maryland, United States of America; 2 The Henry M. Jackson Foundation for the Advancement of Military Medicine, Bethesda, Maryland, United States of America; 3 Department of Health Administration, Florida Atlantic University College of Business, Boca Raton, Florida, United States of America; Universita degli Studi di Firenze, ITALY

## Abstract

**Introduction:**

To examine the relationship between chronic health conditions and out-of-pocket costs (OOPC) and medical debt.

**Methods:**

Secondary data from the 2013 Panel Study of Income Dynamics (PSID) was used. Households whose head of household and spouse (for married households) were 18 to 64 years old were included.

**Results:**

Households with 1 to 3 chronic conditions had higher odds of having any OOPC compared to households with no chronic conditions (AOR 1.74, 95% CI 1.39, 2.17) (*p* < .01). Households with 1 to 3 and 4 or more chronic health conditions were associated with higher odds of having any medical debt (AOR 2.24, 95% CI 1.75 to 2.87; AOR 5.04, 95% CI 3.04 to 8.34) compared to those with no chronic conditions (*p* < 0.01). Similarly, 1 to 3 and 4 or more chronic health conditions was associated with higher amounts of OOPC (Exponentiated Coefficient 1.18, 95% CI 1.03 to 1.36; Exponentiated Coefficient 1.56, 95% CI 1.17 to 2.07) and medical debt (Exponentiated Coefficient 1.69, 95% CI 1.23 to 2.34; Exponentiated Coefficient 2.73, 95% CI 1.19 to 6.25) compared to households with no chronic conditions (*p* < 0.05).

**Conclusions:**

Findings from this study show that the presence of chronic health conditions impose a large financial burden on some households.

## Introduction

The rise in treatment costs and associated cost sharing for individuals and families with chronic health conditions have been found to impose a significant financial burden on families or individuals, even those with health insurance. The financial burden of adverse health events ranges from filing for bankruptcies [1–3] (the extent of which is controversial, for e.g., [1,2,3]) to medical debt [4–8]. Furthermore, uninsured families with medical debt have even depleted their savings and other assets or forgone the consumption of necessities such as food, heat or rent to pay for their medical debt [6, 7].

More specific to our study, there is an emerging literature on the relationship between chronic health conditions and consumer debt in the elderly using data from the Health and Retirement Study (HRS) [9, 10]. While these studies have provided some insight regarding the impact of chronic health conditions on consumer debt, they tend to focus mostly on the elderly population. One exception is the study by Dobkin et al. [1] that examined the impact of hospital admissions on out of pocket spending and unpaid bills for non-elderly adults aged from 50 to 64 years. But, none of these studies focus on specific health adverse events such as chronic health events. Our study investigates the association between chronic health conditions and measures of financial burden in the adult population from 18 to 64 years old. The goal of the current study is to examine the relationship between chronic health conditions and financial burden (out of pocket costs and medical debt) outcomes in the non-elderly population.

This study hypothesized that chronic health conditions would be associated with medical debt outcomes through two major pathways. The first major pathway is through large and repeated out-of-pocket costs (OOPC) of treatment. There is an extensive literature, mostly in the elderly population, that has documented the effect of chronic health conditions on OOPC using data from the HRS or Medicare claims files [11–20]. Similar results have been found among the non-elderly, where about 13% of individuals incurred OOPC exceeding 20% of their annual income [21].

The second major pathway is through the loss of employment. Households that experience chronic health conditions may also simultaneously experience a “productivity” shock [7]. On the one hand, chronic health conditions make it difficult for patients to balance activities such as employment while seeking medical treatment, thereby increasing their taste for leisure. On the other hand, chronic health conditions might encourage labor force participation for those who are able to work because of the link between employment and health insurance in the United States [22]. Most studies in this area tend to focus on the impact of cancer treatment and survivorship on the labor markets’ outcomes of patients [23]. Other chronic conditions such as diabetes and depression have also been found to negatively impact an individual’s ability to participate in the labor markets and can result in a loss of income or reduction in wages [24, 25].

More importantly, as health insurance plays a key role in the relationship between chronic health conditions and medical debt outcomes, it is essential to consider some emerging but well-designed studies in this area that seek to understand to what extent that providing coverage to uninsured patients reduces the financial burden associated with treatment. In this case, four recent studies using data from the Massachusetts Health Insurance Reform of 2006 and the Oregon Health Insurance Experiment of 2008 have found that insurance coverage reduced the share and total amount of debt that was past due, including medical debt for newly insured residents [26–29].

There is limited understanding in the literature on the relationship between chronic health conditions and OOPC and medical debt outcomes at the household level. It is important that decision makers and policy makers are informed of and understand this relationship because of the high financial burden that these conditions might impose on indivdiduals, households and society at large.

## Method

### Data source and subjects

**Fig 1:**
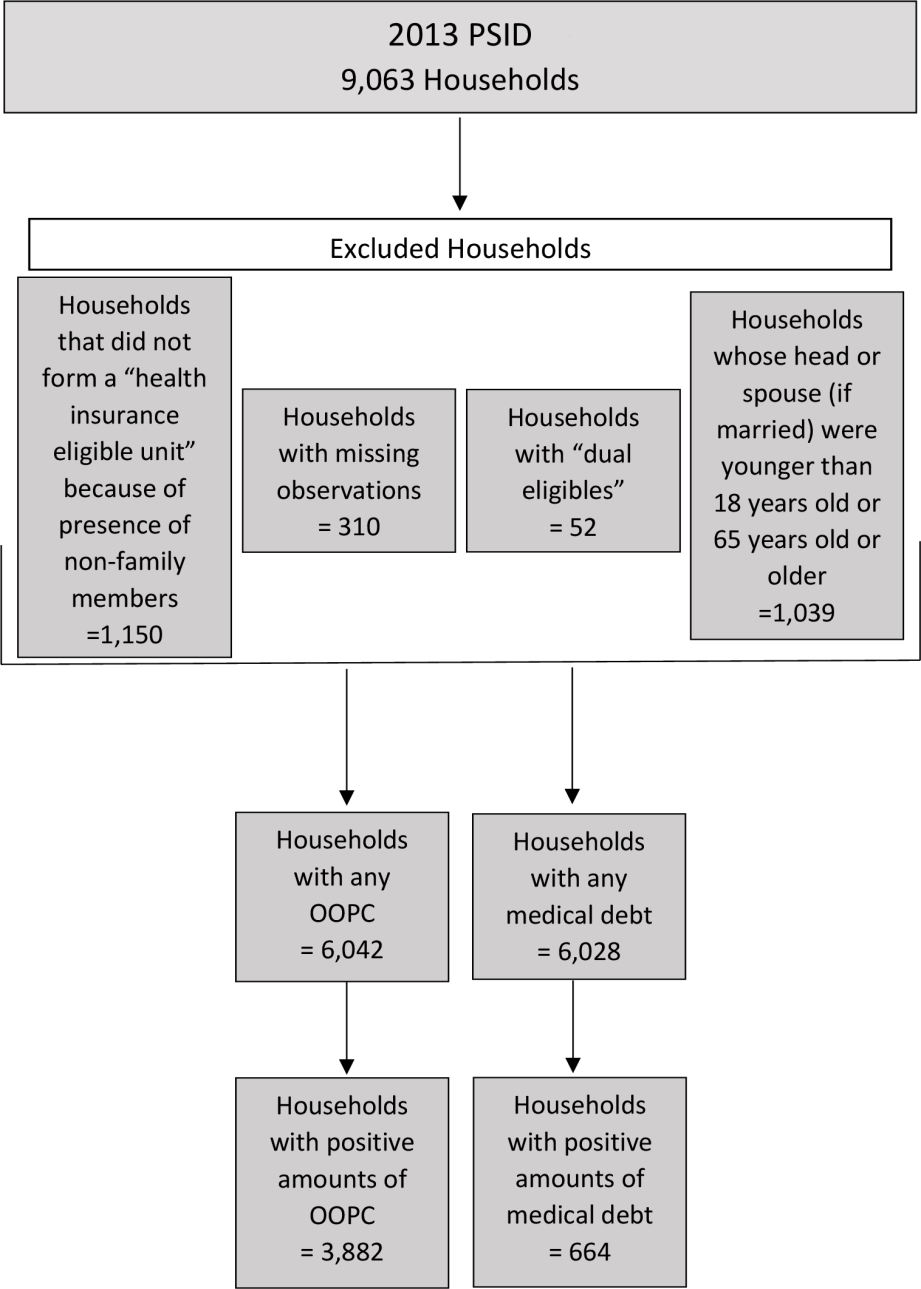
Sample selection process. The figure depicts the number of households in the 2013 PSID followed by the criteria used for exclusion and the number of households excluded, and then the final sample sizes for households with any out-of-pocket costs (OOPC), any medical debt, positive amounts of OOPC and positive amounts of medical debt.

Data from the 2013 Panel Study of Income Dynamics (PSID) was used. The PSID is a nationally representative panel survey that collects socioeconomic and demographic data on households and individuals in the United States. One round of interviews is conducted per panel. The 2013 PSID includes 9,063 households. This study uses households as the unit of analysis. Analyses were done at the household level because the dependent variables are measured at this level. Households whose head of household and spouse (for married households) were 18 to 64 years old were included (see Fig 1). Only households that formed a “health insurance eligible unit” were included (i.e., those with non-family members such as relatives and friends living in the home were excluded). One reason for this is because the head of household is unlikely to incur debt on their behalf. Moreover, including them might bias the results as some of these families might have chronic health conditions of their own. A further exclusion included households whose head or spouse if married was 65 years old or older because of the study’s focus on the non-elderly. “Dual eligibles,” beneficiaries of both Medicare and Medicaid, were also excluded because of unique health needs that may interact with chronic health conditions. Most of the households that were not included were based on these criteria; the remaining, 310 households, had missing observations (given the small number of missing data, we do not believe that it would have an impact on the results). Consistent with the literature in this area, for outcomes of the amount of OOPC and medical debt, only households with positive amounts were included [3, 30–32]. The analytic sample for the outcome of amount of OOPC was 3,882 of the 6,042 households with any OOPC and amount of medical debt was 664 of the 6,028 households with any medical debt.

### Measurement

#### Dependent variables

Dependent variables included: 1) any OOPC, 2) amount of OOPC (OOPC > 0; $2013); 3) any medical debt and; 4) amount of medical debt (medical debt >0; $2013). OOPC was determined by the responses to the following questions: “About how much did (you (and your family)/they) pay out-of-pocket for nursing home and hospital bills in 2011 and 2012 combined?” and “About how much did you (and your family) pay out-of-pocket for doctor, outpatient surgery, and dental bills in 2011 and 2012 combined?” Medical debt was determined by the response to the following: “If you added up all medical bills [respondents/family members living there], about how much would they amount to right now? INCLUDE unpaid balance(s), or medical bills that are outstanding.” Responses are validated by using bills and other documents from the respondents. Several studies have found these measures to be highly reliable [33, 34]. Only those with outstanding medical bills were considered. The National Health Interview Survey measures medical debt similarly [8, 35]. A binary measure was created for any OOPC and any medical debt being coded as 1 if the respondent has any OOPC or medical debt, respectively, or 0 otherwise. Amount of OOPC and amount of medical debt were continuous variables reflecting the amount of OOPC and medical debt, respectively, as indicated by the respondent. Missing values were imputed by the PSID.

#### Independent variables

The key independent variable for this study is chronic conditions that included hypertension or high blood pressure, heart disease, diabetes, asthma, lung disease, cancer, schizophrenia, bipolar disorder, stroke, heart attack, and arthritis was created [36]. For each chronic condition, respondents were asked “Has a doctor or other health professional EVER told (you/HEAD) that (you/he/she) had …CONDITION?”. Based on the distribution of chronic conditions in the study sample, the number of self-reported chronic health conditions was categorized into no chronic conditions, 1 to 3 chronic conditions, and 4 or more chronic conditions. Variables associated with consumer debt or measures of financial burden outcomes were controlled for [10, 26, 28, 29, 37]. The head of household’s gender, age category, race, marital status, and education and household characteristics (both the head and spouse if married) of insurance coverage status, number of chronic health conditions, and number of children were controlled for. Geographic region variables were included to capture differences in health insurance and medical debt outcomes by location. The amount of OOPC, amount of medical debt and number of children were log transformed to adjust for the skewness in the distribution of the variables. In order to account for an oversampling of immigrant and minority households and for attrition, family weights were utilized [38].

### Statistical analysis

Logistic regression models that adjusted for a set of covariates were used to estimate the relationship between chronic health conditions and the likelihood of having any OOPC or medical debt. Odds ratios were presented for these models for better interpretation of the results. Generalized linear models (GLMs) with logarithmic link and a gamma distribution were used to estimate the relationship between chronic health conditions and the amount of OOPC (> 0) or medical debt (> 0) (see [39]). Exponentiated coefficients were presented for these models for better interpretation of the results.

## Results

### Descriptive statistics

The total number of households for the study is 5,860 and 78% of households had any type of debt. For the outcomes of the study, 73% of the households had any OOPC and 9% had any medical debt. The average amount of OOPC was $1171 and the average amount of medical debt was $838.

In terms of analyses, we examined the relationship between chronic health conditions and financial outcomes by categorizing chronic conditions into no chronic conditions, 1 to 3 chronic conditions and 4 or more chronic conditions. For households that had no chronic conditions, 68% had any OOPC, while for those with 1 to 3 chronic conditions, 80% had any OOPC, and for those with 4 or more chronic conditions, 78% had any OOPC (Table 1). These differences were statistically significant between households with no chronic conditions and 1 to 3 chronic conditions (*p* < .01) and households with no chronic conditions and 4 or more chronic conditions (*p* < .05). For amount of OOPC, we found $968.25 for households with no chronic conditions, $1362.94 for households with 1 to 3 chronic conditions, and $1950.91 for households with 4 or more chronic conditions. These differences were statistically significant between households with no chronic conditions and 1 to 3 chronic conditions (*p* < .01) and households with no chronic conditions and 4 or more chronic conditions (*p* < .01). For households that had no chronic conditions, 7% had any medical debt, while for those with 1 to 3 chronic conditions, 12% had any medical debt, and for those with 4 or more chronic conditions, 19% had any medical debt. These differences were statistically significant between households with no chronic conditions and 1 to 3 chronic conditions (*p* < .01) and households with no chronic conditions and 4 or more chronic conditions (*p* < .01). For amount of medical debt, we found $359.48 for households with no chronic conditions, $1,267.06 for households with 1 to 3 chronic conditions, and $2986.68 for households with 4 or more chronic conditions. These differences were statistically significant between households with no chronic conditions and 1 to 3 chronic conditions (*p* < .01) and households with no chronic conditions and 4 or more chronic conditions (*p* < .05).

**Table 1:** Weighted summary statistics of dependent and independent variables (*N* = 5,860), PSID 2013.

Variables	No CHC Means/Proportion	1 to 3 CHC Means/Proportion	4 or more CHC Means/Proportion
OOPC			
Any OOPC	.68	.80	.78
	[0.64, 0.71]	[0.77, 0.82]	[0.70, 0.86]
Amount of OOPC	968.25	1362.94	1950.91
	[855.95, 1080.55]	[1236.45, 1489.42]	[1408.85, 2492.98]
Log Amount of OOPC	4.33	5.35	5.52
	[4.11, 4.55]	[5.14, 5.56]	[4.92, 6.12]
Medical Debt			
Any Medical Debt	.07	.12	.19
	[0.05, 0.08]	[0.10, 0.14]	[0.11, 0.26]
Amount of Medical Debt	359.48	1267.06	2986.68
	[249.29, 469.67]	[848.05, 1686.08]	[610.81, 5362.55]
Log Amount of Medical Debt	0.53	0.96	1.55
	[0.42, 0.64]	[0.82, 1.10]	[0.91, 2.19]
Gender			
Female HOH	.29	.24	.08
	[0.26, 0.31]	[0.21, 0.27]	[0.03, 0.14]
Male HOH	.71	.76	.92
	[0.69, 0.74]	[0.73, 0.79]	[0.86, 0.97]
Age (categories)			
HOH Age 18–34 years	.36	.18	.01
	[0.34, 0.38]	[0.16, 0.20]	[0.00, 0.02]
HOH Age 35–44 years	.24	.21	.09
	[0.22, 0.26]	[0.19, 0.24]	[0.04, 0.14]
HOH Age 45–64 years	.40	.61	.90
	[0.37, 0.43]	[0.58, 0.64]	[0.85, 0.94]
Race			
White HOH	.69	.73	.82
	[0.62, 0.75]	[0.68, 0.78]	[0.74, 0.90]
Black HOH	.17	.15	.11
	[0.12, 0.22]	[0.11, 0.19]	[0.04, 0.17]
Hispanic HOH	.11	.10	.06
	[0.08, 0.15]	[0.07, 0.13]	[0.01, 0.10]
Other	.03	.02	.02
	[0.02, 0.04]	[0.01, 0.03]	[-0.00, 0.04]
Marital Status			
Married	.43	.59	.80
	[0.40, 0.46]	[0.56, 0.62]	[0.74, 0.86]
Never Married	.34	.20	.03
	[0.31, 0.37]	[0.18, 0.23]	[0.00, 0.06]
Not Married++	.23	.21	.17
	[0.21, 0.25]	[0.19, 0.24]	[0.10, 0.23]
Education			
Years of Education	13.92	13.69	12.93
	[13.70, 14.14]	[13.47, 13.92]	[12.23, 13.63]
Years of Education^2^	200.09	194.15	176.57
	[194.48, 205.69]	[188.45, 199.86]	[159.89, 193.25]
Health Insurance Status			
Employer/Private Insurance	.73	.72	.68
	[0.69, 0.76]	[0.69, 0.75]	[0.59, 0.78]
Medicaid	.06	.07	.15
	[0.05, 0.08]	[0.06, 0.08]	[0.08, 0.22]
Uninsured	.21	.20	.16
	[0.18, 0.23]	[0.17, 0.22]	[0.10, 0.23]
Children			
Number of Children	0.82	0.71	0.3
	[0.76, 0.89]	[0.66, 0.77]	[0.18, 0.42]
Log Number of Children	0.44	0.38	0.17
	[0.41, 0.47]	[0.35, 0.41]	[0.10, 0.23]
Geographic Variation			
Northeast	.19	.18	.13
	[0.12, 0.25]	[0.11, 0.26]	[0.03, 0.23]
North Central	.27	.26	.30
	[0.19, 0.35]	[0.19, 0.34]	[0.16, 0.43]
South	.32	.34	.42
	[0.26, 0.38]	[0.27, 0.40]	[0.28, 0.56]
West	.23	.22	.15
	[0.16, 0.29]	[0.14, 0.29]	[0.07, 0.23]
Region			
Urban	.69	.66	.55
	[0.63, 0.74]	[0.60, 0.72]	[0.43, 0.67]
Rural	.31	.34	.45
	[0.26, 0.37]	[0.28, 0.40]	[0.33, 0.57]
Observations	3286	2400	174

Note: 95% confidence intervals are presented in brackets. The number of children and the years of education are continuous variables.

Legend: CHC = Chronic Health Conditions, HOH = Head of Household, Not Married++ = Separated, Divorced or Widowed, Years of Education^2^ = Years of Education Completed Square, OOPC = Out of Pocket Costs.

### Multivariate models

Households with 1 to 3 chronic conditions compared to households with no chronic conditions had 1.74 odds of having any OOPC (95% CI 1.39 to 2.17) (*p* < .01) (Table 2). However, beyond 1 to 3 chronic conditions, there was a non-linear association between households and chronic conditions and the odds of having any OOPC. For instance, while households with 4 or more chronic conditions have higher odds of having any OOPC compared to households with no chronic conditions, the results were not statistically significant. Adjusted odds ratios showed that households in which members (the head and spouse) had 1 to 3 and 4 or more chronic health conditions were associated with higher odds of having any medical debt (AOR 2.24, 95% CI 1.75 to 2.87; AOR 5.04, 95% CI 3.04 to 8.34) compared to those households where members had no chronic conditions (*p* < 0.01). Similarly, exponentiated coefficients from GLM models that used a log link with gamma distribution for households with positive amounts of OOPC and medical debt found that 1 to 3 and 4 or more chronic health conditions were associated with higher amounts of OOPC (Exponentiated Coefficient 1.18, 95% CI 1.03 to 1.36; Exponentiated Coefficient 1.56, 95% CI 1.17 to 2.07) and the amount of medical debt (Exponentiated Coefficient 1.69, 95% CI 1.23 to 2.34; Exponentiated Coefficient 2.73, 95% CI 1.19 to 6.25) compared to households with no chronic conditions (*p* < 0.05). These represent increases over the constant terms of about $174 and $542 in the amount of OOPC for 1 to 3 and 4 or more chronic conditions, respectively and $248 and $622 in the amount of medical debt for 1 to 3 and 4 or more chronic conditions, respectively.

**Table 2:** Results from multivariate models for any out of pocket costs, amount of out of pocket costs (> 0), any medical debt and amount of medical debt (> 0) (95% confidence intervals are in parentheses), PSID 2013.

Variable	Odds Ratios of Any OOPC	Exponentiated Coefficients for Amount of OOPC	Odds Ratios of Any Medical Debt	Exponentiated Coefficients for Amount of Medical Debt
CHC				
1 to 3 CHC	1.74***	1.18**	2.24***	1.69***
	[1.39, 2.17]	[1.03, 1.36]	[1.75, 2.87]	[1.23, 2.34]
4 or more CHC	1.5	1.56***	5.04***	2.73**
	[0.85, 2.63]	[1.17, 2.07]	[3.04, 8.34]	[1.19, 6.25]
Gender (Male HOH)				
Female HOH	1.58***	0.98	1.92***	1.29
	[1.23, 2.03]	[0.77, 1.26]	[1.27, 2.89]	[0.84, 1.96]
Age (categories) (HOH Age 18–34 years)				
HOH Age 35–44 years	1.49***	1.12	0.79	1.29
	[1.17, 1.90]	[0.95, 1.32]	[0.56, 1.13]	[0.91, 1.84]
HOH Age 45–64 years	1.64***	1.36***	0.41***	1.29
	[1.30, 2.06]	[1.13, 1.62]	[0.29, 0.58]	[0.89, 1.87]
Race (White HOH)				
Black HOH	0.42***	0.75***	0.97	1.40*
	[0.33, 0.54]	[0.60, 0.92]	[0.68, 1.38]	[0.96, 2.05]
Hispanic HOH	0.70**	0.70***	0.77	0.72
	[0.51, 0.97]	[0.58, 0.84]	[0.51, 1.17]	[0.43, 1.22]
Other	0.63*	1.2	0.78	3.18*
	[0.37, 1.06]	[0.76, 1.91]	[0.33, 1.81]	[0.88, 11.48]
Marital Status (Married)				
Never Married	0.35***	0.65***	0.85	0.8
	[0.27, 0.45]	[0.52, 0.80]	[0.57, 1.28]	[0.48, 1.35]
Not Married++	0.39***	0.80**	1.03	1.1
	[0.29, 0.54]	[0.66, 0.98]	[0.64, 1.68]	[0.69, 1.74]
Education				
Years of Education	1.04	0.91	1.29	0.95
	[0.88, 1.24]	[0.80, 1.04]	[0.93, 1.79]	[0.78, 1.16]
Years of Education^2^	1.00	1.00*	0.98**	1.00
	[0.99, 1.01]	[1.00, 1.01]	[0.97, 1.00]	[0.99, 1.01]
Health Insurance Status (Employer/Private Insurance)				
Medicaid	0.13***	0.58***	0.66*	1.64*
	[0.10, 0.19]	[0.42, 0.82]	[0.42, 1.02]	[0.94, 2.85]
Uninsured	0.32***	1.20*	1.32	2.32***
	[0.24, 0.41]	[0.98, 1.47]	[0.95, 1.84]	[1.58, 3.40]
Children				
Log Number of Children	1.04	1.33***	1.11	1.30*
	[0.85, 1.26]	[1.18, 1.50]	[0.87, 1.40]	[0.97, 1.74]
Geographic Region (Northeast)				
North Central	0.86	1.1	1.66***	1.97**
	[0.64, 1.17]	[0.88, 1.39]	[1.15, 2.40]	[1.06, 3.69]
South	0.93	1.05	1.83***	1.52
	[0.70, 1.24]	[0.85, 1.31]	[1.28, 2.61]	[0.84, 2.75]
West	0.92	1.07	1.47	1.14
	[0.66, 1.27]	[0.84, 1.35]	[0.91, 2.38]	[0.62, 2.10]
Region (Rural)				
Urban	1.06	0.99	0.75***	1.04
	[0.84, 1.33]	[0.86, 1.13]	[0.62, 0.92]	[0.74, 1.47]
Observations	6,042	3,882	6,028	664

*p < .10

**p < .05

***p < .01

Legend: CHC = Chronic Health Conditions, HOH = Head of Household, Not Married++ = Separated, Divorced or Widowed, Years of Education^2^ = Years of Education Completed Square, OOPC = Out of Pocket Costs. Reference groups are in parentheses.

Note: The difference in sample size between Tables 1 and 2 is from including variables that were not log transformed in Table 1 (e.g., number of CHC) and these were not in the analysis in Table 2, resulting in some variables not having weights.

Additionally, households that were headed by females were more likely to have any OOPC or any medical debt compared to households that were headed by males. Interestingly, households headed by those who were between 45 and 64 years old were more likely to have any OOPC but less likely to have any medical debt compared to those where the head was between 18 and 34 years old. Medicaid beneficiary households were less likely to have any OOPC compared to those that were privately insured. Uninsured households were less likely to have any OOPC compared to privately insured households. However, for those with any OOPC, uninsured households were more likely to have higher amounts of OOPC compared to those with private health insurance. These findings are consistent with similar studies in the literature [40, 41], which reflect issues of access to health care by those who do not have health insurance, but experience higher amounts of OOPC when accessing health services compared to those who are privately insured. Those who live in the South and the Midwest were more likely to have any medical debt compared to those who live in the northeastern region of the United States. These may reflect the variations in health insurance across the different regions of the United States. In terms of amount, households headed by 45 to 64 year olds were associated with higher amounts of OOPC compared to those headed by individuals who were between 18 and 34 years old. Similarly, households that lacked health insurance or headed by Medicaid beneficiaries were associated with a higher amount of medical debt compared to those with private insurance.

## Discussion

Findings showed that 1 to 3 chronic health conditions were associated with higher odds of having any OOPC and 1 to 3 and 4 or more chronic health conditions were associated with higher odds of having any medical debt at the household level. Similarly, for households with positive OOPC and medical debt, 1 to 3 and 4 or more chronic health conditions were associated with higher amounts of OOPC and medical debt, representing increases over the constant terms of about $174 and $542 in the amount of OOPC for 1 to 3 and 4 or more chronic conditions, respectively and $248 and $622 in the amount of medical debt for 1 to 3 and 4 or more chronic conditions, respectively. Findings from our study are consistent with those of the studies conducted by Kim et al. (2012) and Barbiarz et al. (2013) [9, 10] that have looked at the association between adverse health events and unsecured debt using data from the HRS. Defining a new health event as those households where the head has been diagnosed with any of the following conditions including high blood pressure, diabetes, cancer, lung disease, heart problems, stroke, arthritis, or psychological problems, Barbiarz et al. (2013) [9, 10] found that new health events are associated with a 10% increase in the probability of having any unsecured debt and approximately an 11% increase in the amount of unsecured debt. Converting the odds ratios from the logistic regressions into marginal effects by using the marginal effects command from STATA 14 (results not presented) and the marginal effects for regression coefficients for dummy variables calculated as *e^x^* − 1 (see Table 2) [3, 42], we found that a change by household members from having no chronic conditions to having 1 to 3 chronic conditions was associated with a change in the probability of having any OOPC by 15% and any medical debt by 84%. Further, a change by household members from having no chronic conditions to having 4 or more chronic conditions was associated with a change in the probability of having any medical debt by 168%. However, the exponentiated coefficients from Table 2 (columns 2 and 4) showed that 1 to 3 and 4 or more chronic health conditions were associated with increased amounts of OOPC by 18% and 56% and medical debt by 69% and 173% for those households with positive amounts of OOPC and medical debt. These results are much greater in magnitude compared to those found by Kim et al. (2012), Barbiarz et al. (2013), and Dobkin et al. [9, 10]. There are several plausible explanations for these differences in the amounts of OOPC and medical debt including the fact that our sample has a higher proportion of uninsured households, examines medical debt as opposed to unsecured debt and considers OOPC for patients in inpatient hospital services whether the head or spouse experiences the chronic health condition in the household. Additionally, contrary to Dobkin et al. [1] whose study used a sample of patients admitted to hospitals in California, our study used broader measures of OOPC and medical debt that included nursing homes, hospital bills, ambulatory care and dental care. It is important to examine the relationship between chronic conditions and measures of financial burden for insured and uninsured households aged 18 and 64 years old to understand how these conditions might impact different age groups and households by types of insurance status differently.

There are some limitations that are worth considering in this study. Although a high proportion of the medical debt amount was validated by the PSID through verification of the actual medical bills [38], the chronic health conditions were self-reported. Further, it is also important to note that different types of chronic conditions such as cancer or stroke may have a differential association with OOPC and medical debt outcomes compared to other illnesses such as bipolar disorder or asthma. Along similar lines, the severity of the illness and details on the management of the illness were not known.

The findings from this study can be explored in the context of public policy, such as in the Affordable Care Act (ACA). State and federal policy changes to decrease the financial burden of households with chronic health conditions have recently occurred [43]. Nonetheless, findings from the current study show that chronic health conditions still have a financial burden on some households. Additionally, the study results can inform medical professionals about the financial impact of clinical decisions and treatments for patients with chronic health conditions.

The essential benefits coverage provisions of health services from the ACA have important implications for households whose chronic health conditions are associated with medical debt or OOPC. For example, certain preventive services must be included without cost-sharing. An increase in preventive services could decrease inpatient hospital services for chronic health conditions while decreasing the household’s financial burden. Further research might want to consider the impact of the ACA on medical debt and OOPC for households where members have experienced chronic health conditions.

## Conclusion

Findings showed that 1 to 3 chronic health conditions were associated with higher odds of having any OOPC and 1 to 3 and 4 or more chronic health conditions were associated with higher odds of having any medical debt. For households with positive amounts of OOPC and medical debt, 1 to 3 and 4 or more chronic health conditions were associated with higher amounts of OOPC and medical debt. These findings showed that the presence of chronic health conditions impose a large financial burden on some households. An important extension of this paper would be to use instrumental variables to account for any potential omitted variable bias or reverse causality. Future research may also want to look at longitudinal data to assess the impact of chronic conditions on medical debt and OOPC.
